# The Role of Drying Schedule and Conditioning in Moisture Uniformity in Wood: A Machine Learning Approach

**DOI:** 10.3390/polym15040792

**Published:** 2023-02-04

**Authors:** Sohrab Rahimi, Vahid Nasir, Stavros Avramidis, Farrokh Sassani

**Affiliations:** 1 Department of Wood Science, University of British Columbia, Vancouver, BC V6T 1Z4, Canada; 2 FPInnovations, Vancouver, BC V6T 1Z4, Canada; 3 Department of Mechanical Engineering, University of British Columbia, Vancouver, BC V6T 1Z4, Canada

**Keywords:** western hemlock, wood moisture, drying schedule, conditioning, TreeNet gradient-boosting, machine learning, ensemble learning

## Abstract

Monitoring the moisture content (MC) of wood and avoiding large MC variation is a crucial task as a large moisture spread after drying significantly devalues the product, especially in species with high green MC spread. Therefore, this research aims to optimize kiln-drying and provides a predictive approach to estimate and classify target timber moisture, using a gradient-boosting machine learning model. Inputs include three wood attributes (initial moisture, initial weight, and basic density) and three drying parameters (schedule, conditioning, and post-storage). Results show that initial weight has the highest correlation with the final moisture and possesses the highest relative importance in both predictive and classifier models. This model demonstrated a drop in training accuracy after removing schedule, conditioning, and post-storage from inputs, emphasizing that the drying parameters are significant in the robustness of the model. However, the regression-based model failed to satisfactorily predict the moisture after kiln-drying. In contrast, the classifying model is capable of classifying dried wood into acceptable, over-, and under-dried groups, which could apply to timber pre- and post-sorting. Overall, the gradient-boosting model successfully classified the moisture in kiln-dried western hemlock timber.

## 1. Introduction

Timber drying impacts the final product quality and plays an essential role in the dimensional stability, and mechanical properties of timber, as the physical [1], elastic and viscoelastic properties of wood are moisture-dependent [2]. Additionally, timber drying facilitates coating, cutting, and remanufacturing procedures, rendering dried wood less susceptible to deformation, cracks, and decay [3,4]. Target moisture (*M_t_)* in kiln-drying should correspond to the indoor or outdoor environment where the final product will be used. A well-managed drying operation dramatically improves timber drying quality [5,6].

Considerable variability in initial moisture (*M_i_)* among timber pieces of the same kiln batch is inevitable, especially in softwoods with substantial differences between the properties of sapwood and heartwood [7]. Different timber pieces in the same load undergo various drying levels due to inherent variations in *M_i_* and other wood properties [8,9]. Consequently, final moisture (*M_f_*) among timber fluctuates along a broad range. Large-scale kilns aggravate this problem because of the non-uniform kiln-drying conditions in the chamber’s width, depth, and height [10]. Non-uniformity of the final moisture between kiln-dried timber pieces substantially impacts their monetary values. Over-dried wood is in less demand in the market since over-drying increases shrinkage and shapes distortion [11]. Additionally, under-dried wood is hardly acceptable in the market due to its susceptibility to fungal decay and low mechanical strength [11].

Almost every wood species requires a specific drying schedule, which could be time-based, moisture-based, or combined [12]. Drying schedules involve predetermined heat, humidification, ventilation, and air circulation [13,14]. Combined (time- and moisture-based) schedules typically apply to the kiln-drying coastal softwood species in British Columbia, Canada. In a combined schedule, drying factors are time-based from the beginning of the drying until around the fiber saturation point (*M_fsp_*). However, upon reaching that point, they become constant until reaching *M_t_*. Drying schedules’ aggressiveness influences the final moisture variation in a kiln batch [8,15]. Moreover, post-drying steps such as conditioning in kilns and outdoor storage may apply to reduce moisture variation within and between timber pieces [16,17]. Conditioning is a high-humidity step at the end of some drying schedules (after reaching *M_t_*) to minimize the moisture differences between and within timber pieces (shell and core of timber) and relieve internal stresses (casehardening) [9]. However, some Japanese sawmills use stickers and store kiln-dried batches outdoors for a period of one to two weeks to reduce the moisture variation and moisture profile in thickness, resulting in internal stress dissipation [16].

Pacific coast hemlock (also known as ‘‘hem-fir’’) is an abundant source of fiber on the British Columbia coast which is comprised of western hemlock (*Tsuga heterophylla*) and amabilis fir (*Abies amabilis*) [18]. Thick solid hem-fir products, specifically timbers with cross-sectional areas of 90 × 90 mm^2^, 105 × 105 mm^2^, and 115 × 115 mm^2^ (also known as “baby-squares”) are commonly the preferred material in timber construction, especially in Japan, which is one of BC’s largest overseas markets [19]. Hemlock is a difficult-to-dry species due to its naturally high green moisture content, the presence of wet wood (or wet pockets), and often compression wood. Past research focused on optimizing drying schedules [15,20,21], pre-sorting [22,23,24], and post-sorting [25] strategies. Studies examining the collapse and recovery during the drying process [26,27], cracking occurrence during the drying process [28], and numerical simulations of coupled moisture and heat transfer in wood during kiln drying [29] were also reported in the literature. Kiln-drying scheduling is also covered in some studies [30,31] as an important factor impacting the final moisture content and drying defects [32]. In addition, previous studies focused on characterizing and modeling final moisture and its spread in air-dried [33], radio-frequency kiln-dried [34,35,36], heat treated [37], and heat-and-vent kiln-dried batches [38,39,40]. Additionally, previous studies investigated moisture prediction in kiln-dried lumber merely based on wood properties; however, the combined effects of drying conditions and wood properties on the moisture uniformity after kiln-drying still represent a knowledge gap [41]. Furthermore, initial wood indices, especially *M_i_* content and its variation, remarkably affect *M_f_* variation. Therefore, a holistic approach is required to characterize the combined effects of wood indices and drying schedules on wood properties after kiln-drying [38]. For this reason, the current study aims to investigate and predict the *M_f_* of kiln baby-square western hemlock under different schedules.

Accordingly, a machine learning approach was adopted to study the relationship between the wood properties and to quantify the roles of drying schedule, conditioning, and post-storage. This study uses a gradient-boosting algorithm known as TreeNet for moisture prediction and classification. The most widely used machine learning models in the literature on wood science and technology are artificial neural networks (ANNs). They have been used in a wide range of applications for wood identification [42,43], defect detection [42,43], and wood properties prediction [44,45]. The most emphases were on employing the multilayer perceptron (MLP) model [46,47,48]. However, compared to ANNs, fewer studies investigated the performance of ensemble machine learning methods such as gradient boosting for predicting wood properties. Ensemble learning improves prediction accuracy by using multiple machine learning algorithms known as a weak learner and fusing the results by applying a different voting mechanism [49]. This study uses the TreeNet gradient boosting model, variable clustering, and correlation analysis to predict the *M_f_* in kiln-dried western hemlock and explain the role of initial wood properties, drying schedules, and conditioning on the moisture distribution in dried timber.

## 2. Materials and Methods

### 2.1. Materials

**Figure 1:**
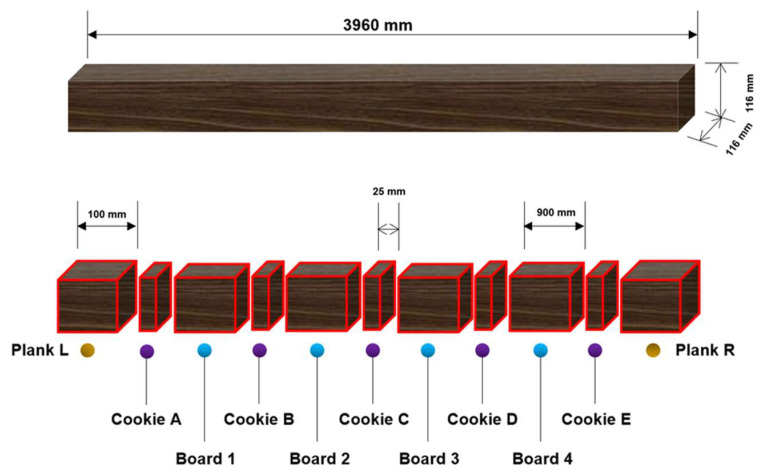
Cutting pattern of the baby-square western hemlock.

A local sawmill located on Vancouver Island, British Columbia provided 96 timber pieces of second-growth western hemlock baby squares (116 mm × 116 mm; 3.96 m in length) for this study. All timber pieces were in green condition with a grade of II (standard) or better [50]. Each piece was cut into four kiln specimens and five cookies using a circular saw. Figure 1 represents the cutting protocol. According to the cutting protocol, one section of 100 mm in length was removed from each end of every timber piece to mitigate the risk of end moisture loss. Subsequently, four kiln specimens and five cookies were cut from each timber piece. The length of the kiln specimens and cookies were 900 mm and 25 mm, respectively. Overall, 480 cookies and 384 kiln specimens were provided from the entire timber population.

### 2.2. Experiments

Cookies were used to measure *M_i_* and basic density (*ρ_b_*) according to Kollmann [51] and Skaar [52]. In the next step, six out of 384 kiln specimens were arbitrarily discarded, and the rest (378 kiln specimens) were randomly assigned to nine drying batches. Table 1 summarizes the nine drying runs used in this study. The control drying schedule was applied to the first drying batch, followed by conditioning. The first modified drying schedule had four modes as the combination of presence and absence of conditioning and post-storage. Similarly, the second modified drying schedule had four modes as the combination of existence and nonexistence of conditioning and post-storage.

Each drying batch contained 42 timber specimens. The *M_i_* and *ρ_b_* could influence the *M_f_* and needed to be neutralized to make the drying results comparable between all drying batches. Therefore, the entire timber population was categorized into nine groups so that *M_i_* and *ρ_b_* had the smallest standard deviation. The cross-sections of the specimens were coated using polyvinyl acetate (PVA) before drying to prevent end moisture loss. A conventional heat-and-vent kiln with a capacity of 0.73 m^3^ in FPInnovations, Vancouver, British Columbia was used for this research. The same aluminum stickers, with a weight and length of 8.94 kg and 19 mm, respectively, were used for each drying run, which contained 42 kiln specimens (six rows and seven columns).

The drying schedule developed in the past [53,54] was used as the control (unmodified) schedule. This time-based schedule consisted of eight steps, using a pre-determined number of hours for each step. In step nine, the drying process was switched to a moisture content-based schedule, drying the timber to the *M_t_* without changing the settings. The *M_t_* was set to 12%, the average equilibrium moisture content *M_emc_* in Japan from October to May [16]. This *M_t_* was chosen to avoid additional moisture loss from the specimens during post-drying storage time. The last step (conditioning) was time-based. After completing a drying run, the timber pieces cooled down for twelve hours inside the kiln with the doors closed. In addition to the control schedule, two modified drying schedules were also used. In schedule I, the same dry-bulb temperature was reached in the last step and the *M_emc_* decreased more aggressively. Schedule II was considered an aggressive drying schedule because it reached a higher dry-bulb temperature in the final drying step, having a steep reduction in *M_emc_*. Furthermore, the *M_f_* was kept under 93 °C to avoid developing a honeycomb. Table 2, Table 3 and Table 4 illustrate the control schedule, schedule I, and schedule II, respectively. All kiln specimens were reweighed post-drying to evaluate their final moisture *M_f_*. 

After kiln-drying, all timber pieces were reweighed. The kiln-dried weight or final weight (*w_f_*) of each sample was used to calculate its *M_f_*, according to the equations documented in Perre [8] and Siau [55].

### 2.3. Machine Learning

The model inputs included *M_i_*, *w_i_*, *ρ_b_*, types of drying schedule (control, I, II), conditioning (Yes/No), and post-drying storage (Yes/No). The objective was to predict the *M_f_* and classify the timber condition after drying. Accordingly, the boards with *M_f_ <* 10 and *M_f_ ≥* 19 were labeled as over-dried and under-dried, respectively. Additionally, boards with 10 ≤ *M_f_* < 19 were labeled as normal. TreeNet, a gradient-boosting algorithm, was used for both the regression and classification tasks. It uses the decision tree-based CART model [56] for ensemble learning. Decision tree models are easy to interpret, and the importance of the predictor variables and their relationships can be identified through exploratory data analysis. Details of the CART model can be found elsewhere [57,58]. The CART algorithm was successfully used for check prediction in weathered thermally modified timber [59] and for characterization and classification of artificially weathered wood [60,61,62]. Ensemble learning based on bagging or boosting algorithms could be applied to reduce the variance of a single prediction by a tree using multiple weak learners (decision tree). A benchmark study on medium-sized data has shown that tree-based ensemble models such as XGBoost (eXtreme Gradient Boosting) and random forest could outperform the ANNs despite the presence of irregular patterns in the target function and uninformative features [63]. Random forest uses the bagging method, in which each tree is trained using a subset of data, and the model output is based on the voting scheme among weak learners [64]. Random forest was used to predict the mechanical properties of wood fiber insulation boards [65]. It is also utilized in wood machining for tool temperature prediction [66] and frozen lumber classification [67].

Unlike bagging methods, in which weak learners are trained in a similar way, boosting methods perform the training process sequentially, whereas subsequent models correct the performance of prior models. In the gradient-boosting algorithm of TreeNet, a subset of data is used to train a CART model with a maximum number of terminal nodes or tree depth. Then, the CART model is updated depending on the loss function but shrinks the update by the defined learning rate. The process is repeated, and CART models are sequentially added for a specified number of iterations, equal to the number of trees to build [68]. Boosting methods are used for wood species recognition [69], online color classification systems of solid wood flooring [70], predicting the mechanical properties of wood composite [71], and wood machining monitoring [72]. In this study, the number of trees was set to 2000. Additionally, the maximum terminal node per tree and the minimum number of cases allowed for a tree were set to 12 and 3, respectively. Additionally, the learning rate and subsample fraction were equal to 0.01 and 0.3, respectively. Finally, the number of predictors for node splitting was equal to the square root of the total number of predictors.

## 3. Results and Discussion

The results will analyze and characterize the selected initial and final wood indices and their correlation with drying parameters, drying schedule aggressiveness, drying condition, and post-drying storage. Then, a predictive approach will be provided to estimate the *M_f_* of each timber piece based on its corresponding wood properties and drying conditions. Finally, a classification approach will be delivered to categorize dried wood into three groups: Acceptable (normal), over-dried, and under-dried.

### 3.1. Wood Indices and Drying Parameters Analysis

**Figure 2:**
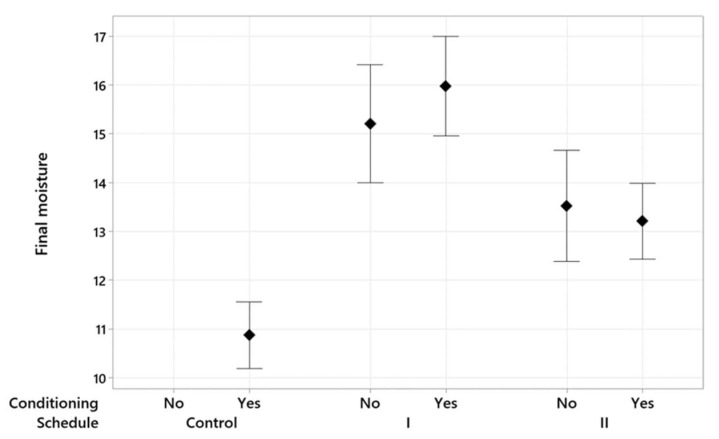
Interval plot of the *M_f_* for different drying schedules with the presence and absence of conditioning treatment. The results are at 95% of the confidence interval for the *M_f_* mean.

**Figure 3:**
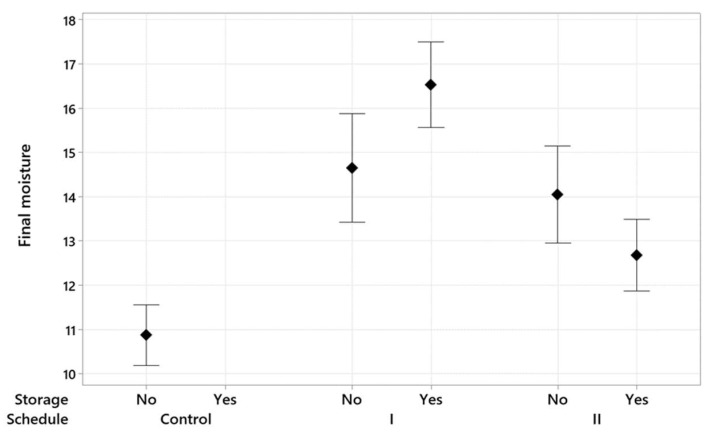
Interval plot of the *M_f_* for different drying schedules with the presence and absence of post-storge treatment. The results are at 95% of the confidence interval for the *M_f_* mean.

Figure 2 and Figure 3 are interval plots representing the impact of conditioning and post-storage on the *M_f_* variation, respectively. These results are based on the 95% of confidence interval for the *M_f_* mean. Both figures indicate that modified drying schedules considerably increased the average *M_f_*, and this effect is more noticeable than the conditioning or post-storage. The control drying schedule had eight time-based steps that took 180 h, which was longer than the modified drying schedules. This long drying time gives grounds to the lower *M_f_* mean at the end of the control drying run, as it allocated sufficient time for under-dried wood to decrease in moisture. Applying conditioning and post-storage reduced the variation in *M_f_* for both modified schedules because, while conditioning and post-storage allow under-dried wood to lose moisture, they let over-dried wood regain moisture. Additionally, while for each drying schedule the role of conditioning and post-storage is insignificant, there was a remarkable difference between the *M_f_* in the two modified drying schedules when the timber pieces underwent conditioning or post-storage.

**Figure 4:**
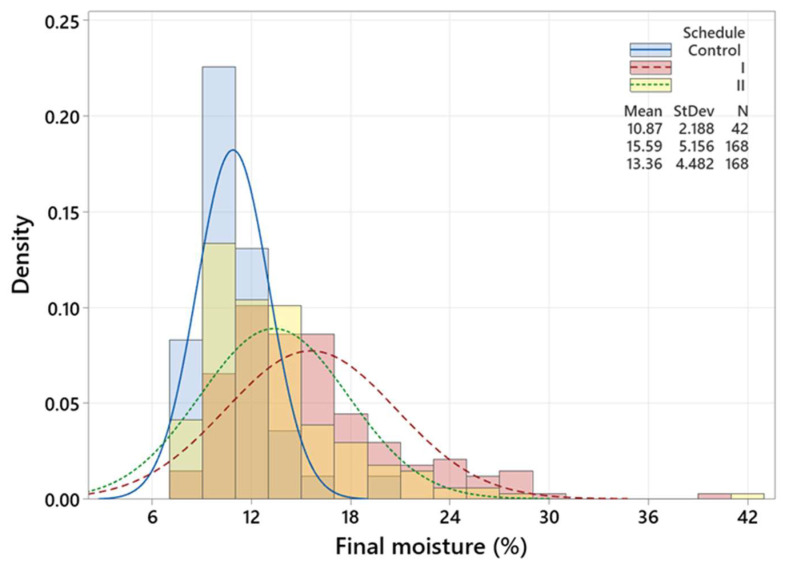
Histogram and distribution curves of the *M_f_* for the control, modified I and modified II schedules.

**Figure 5:**
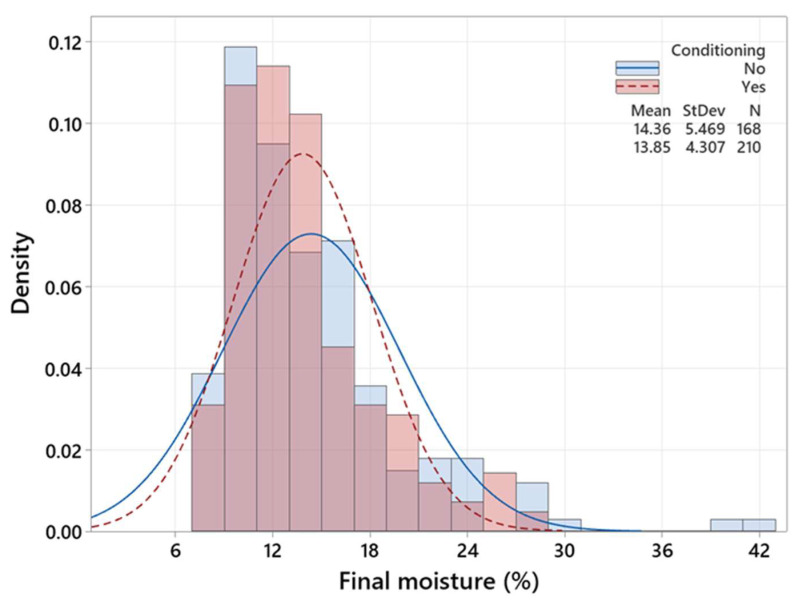
Histogram and distribution curves of the *M_f_* in the existence and nonexistence of the conditioning treatment.

**Figure 6:**
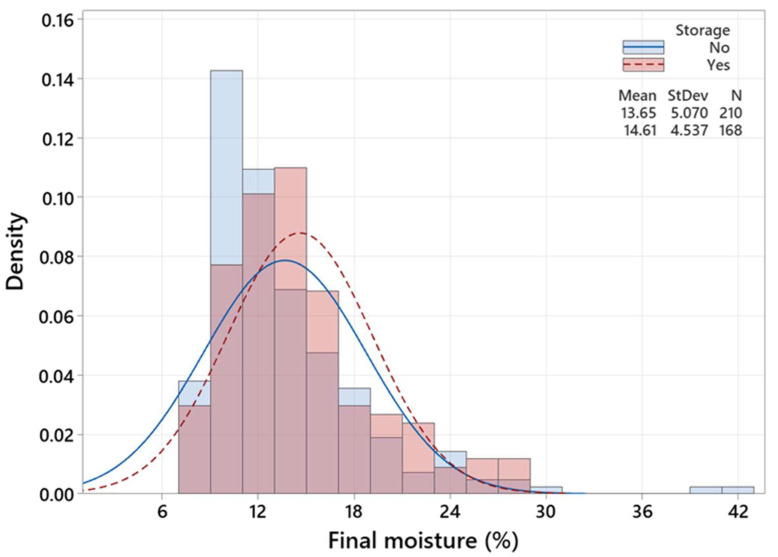
Histogram and distribution curves of the *M_f_* in the existence and nonexistence of the post-storage treatment.

Figure 4, Figure 5 and Figure 6 are three histograms depicting the distribution of *M_f_*. Figure 4 shows that schedule I accounts for the highest *M_f_* mean (15.59%) and variation (5.16%), while the lowest *M_f_* mean (10.87) and variation (2.19) values belong to the 42 timber pieces that underwent the control schedule. The *M_f_* mean is very close to the *M_t_*, which could be attributed to the long drying time compared to the modified ones. Figure 5 demonstrates that timber pieces with conditioning had a slightly smaller standard deviation (4.31%) than those without conditioning (StDev = 5.47%). Likewise, Figure 6 exhibits that timber pieces with post-storage had an insubstantial smaller standard deviation (4.54%) than those without conditioning (StDev = 5.07%). In conditioning and post-storage, the *M_emc_* (12.3%) is very close to *M_t_* (12%), letting the *M_f_* reach *M_t_* and increasing moisture uniformity.

**Figure 7:**
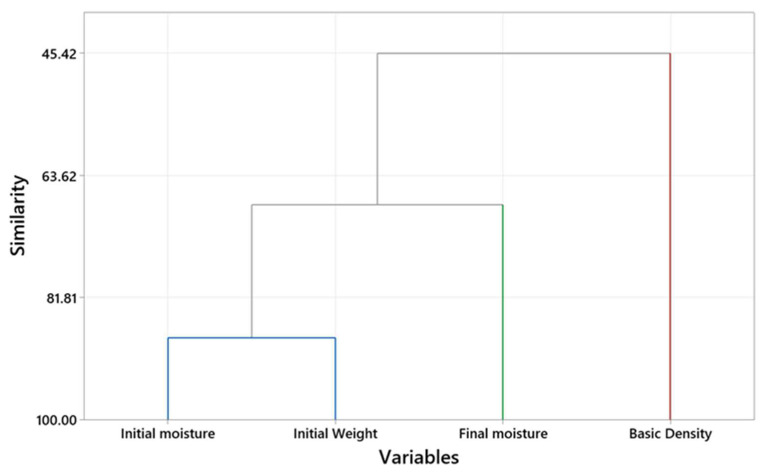
The variable clustering analysis illustrates the correlation coefficient distance between wood attributes.

The dependency between the *w_i_* and *M_i_*, *M_f_*, and *ρ_b_* could be studied through a hierarchical clustering analysis, as explained by Fathi et al. [73]. The dendrogram (Figure 7) demonstrates three clusters and shows the similarity level between the studied variables. This dendrogram indicates that *ρ_b_* had the smallest similarity value (45.42%) with the *M_f_*, which accords with the findings of the previous research on 2″ × 4″ hem-fir [38]. In the present study, *M_i_* and *w_i_* showed the most similarity, while Rahimi and Avramidis [38] observed the most similarity between *M_f_* and *M_i_* in the previous research. The initial weight of the timber can be measured accurately and non-destructively. It is challenging to measure the moisture above the fiber saturation by moisture meters, and cutting cookies is a time-consuming and destructive method that cannot be performed at sawmills. 

It was interesting to see that the drying schedule, conditioning, and post-storage protocols noticeably impacted the correlation between the *M_f_* and the input variables. Table 5 documents the correlation between *M_f_* and initial wood indices in nine drying runs. Accordingly, the highest correlation between *M_i_* and *M_f_* (0.62%) was in I_C_NS (abbreviated names defined in Table 1), while the lowest correlation between *M_i_* and *M_f_* (0.15%) was in UN. Furthermore, the highest correlation between *ρ_b_* and *M_f_* (0.60%) was in II_NC_NS, while the lowest correlation between *ρ_b_* and *M_f_* (0.12%) was in II_NC_S. Moreover, the highest correlation between *w_i_* and *M_f_* (0.75%) was in I_NC_S, whereas the lowest correlation between *w_i_* and *M_f_* (0.38%) was in II_NC_S. Overall, *w_i_* showed the highest correlation values with *M_f_* in all drying runs, excluding II_NC_NS. Overall, *ρ_b_* has the lowest correlation with *M_f_* because *ρ_b_* is naturally based on oven-dried weight and is independent of moisture level. Moreover, the volume change is negligible compared to weight change after kiln-drying, which further justifies the insubstantial correlation between *ρ_b_* and *M_f_*.

However, the correlation values in this study were considerably smaller than the findings of the former study [38]. In the former study, six drying batches underwent an identical drying schedule, while conditioning and post-storage were nonexistent. In contrast, this study included three drying schedules (with different *M_emc_* at the final step) followed by conditioning and post-storage. These two post-drying treatments level out *M_f_* variation and give grounds to the lower correlation between *M_i_* and *M_f_*.

### 3.2. Moisture Prediction by TreeNet

**Figure 8:**
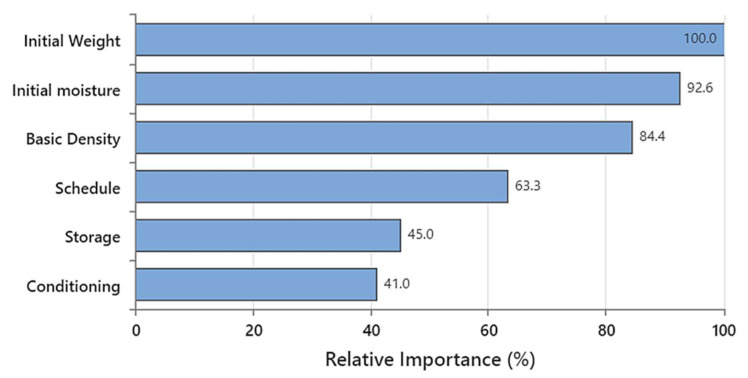
The relative importance of the inputs used to train the random forest approach for the *M_f_* predictive model.

Figure 8 shows the relative importance (RI) of the inputs in the predictive model, indicating that *w_i_* is the most remarkable parameter in this model (RI = 100), followed by the *M_i_* (92.6%) and *ρ_b_* (84.4%). The RI of drying schedule, post-storage, and conditioning were 63.3%, 45.0%, and 41.0%, respectively. This outcome shows that all the listed parameters considerably impact the model’s performance, though they have different RI values. It is worth mentioning that these results are moderately different from the findings by Rahimi et al. [40], in which *M_i_* was the most important input. This difference may stem from different drying schedules, applying post-drying treatments, or different timber dimensions (2″ × 4″ vs. 4″ × 4″).

**Figure 9:**
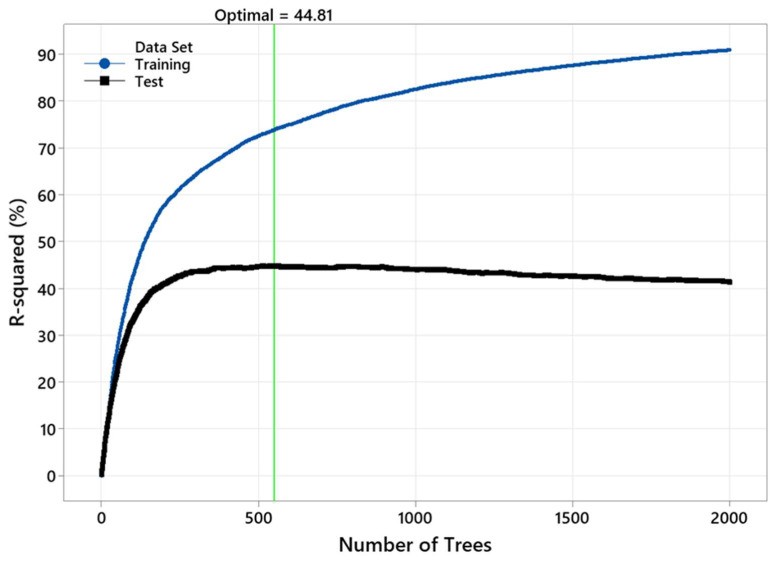
The variation in R^2^ with the number of trees in the TreeNet model.

Table 6 lists the selected statistical parameters for the training and test datasets in the *M_f_* predictive model. These results were based on the six predictors, including three wood attributes (*M_i_*, *w_i_*, and *ρ_b_*) and three drying parameters (schedule, conditioning, and post-storage). The optimal performance (Figure 9) was achieved by having 550 trees in the model. The predictive model had an R^2^ of 73.86% and 44.81% for the training and test, respectively.

**Figure 10:**
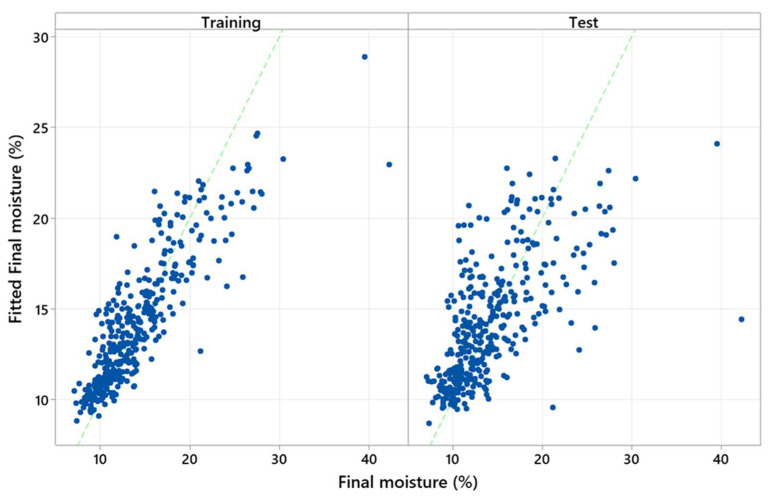
The actual *M_f_* against the predicted *M_f_*.

Figure 9 shows that the R^2^ depends on the number of trees in the TreeNet model. This model had an unsatisfactory performance, with a low number of trees (N < 250). Comparing the training test results in Figure 9 discloses overfitting issues with the model. The actual (experimental) *M_f_* versus the fitted (predicted) *M_f_* is also shown in Figure 10.

An additional study was performed to assess the role of the categorical parameters, including the drying schedule, conditioning, and post-storage, on the performance of the predictive model. Thus, another regression model was trained, using only three parameters (*M_i_*, *w_i_*, and *ρ_b_*). Table 7 shows the model summary for this analysis. This model included 2000 grown trees with 214 optimal numbers of trees. The predictive model had an R^2^ of 56.80% and 32.10% for the training and test, respectively. A comparison between the results of the two models (Table 6 and Table 7) reveals that including the drying parameters improved the accuracy of the predictive model. Overall, the developed model failed to predict the *M_f_* accurately. This failure is justified by the small sample size per drying run (42 boards) compared to the previous research (384 boards) [38]. Furthermore, applying post-drying treatments leveled off the moisture variation and slightly diluted the role of *M_i_* in predicting *M_f_*.

### 3.3. Moisture Classification

**Figure 11:**
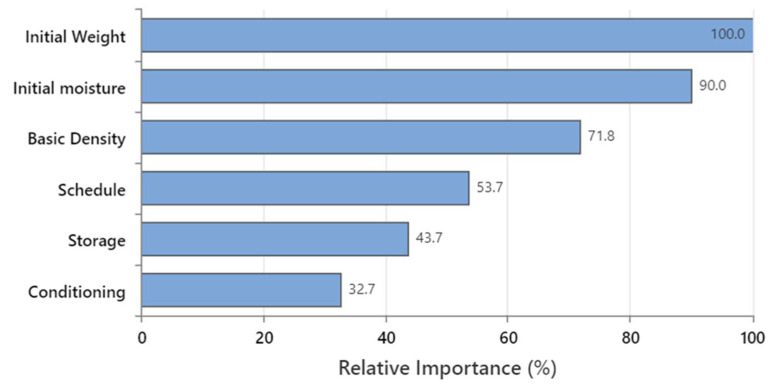
The relative importance of the inputs used to train the random forest approach for the *M_f_* classifier model.

Since the regression approach failed to predict the *M_f_* with acceptable accuracy, it was attempted to classify the *M_f_* as having the input parameters and predict the chance of having over- or under-dried timber. This would be a crucial quality control task for drying processes. Accordingly, the classification was performed using TreeNet with the same assumption defined for the regression approach. Figure 11 illustrates the RI of the inputs in the predictive model for moisture classification. In this model, *w_i_* is the most important parameter (RI = 100), followed by *M_i_* (90.0%) and *ρ_b_* (71.8%). The RI values of schedule, post-storage, and conditioning were, in turn, 53.7%, 43.7%, and 32.7%, respectively. It is observed that the inputs have the same order in terms of relative importance for the moisture prediction (Figure 8) and moisture classification (Figure 11) models.

Table 8 lists the confusion matrix and the classification summary. The highest accuracy for the training and test data belonged to the over-dried (89.66%) and acceptable class (71.64%). Additionally, the lowest accuracy for the training and test data belonged to the acceptable (71.64%) and over-dried (58.62%) classes. Overall, the model could classify the wood with an accuracy of 76.19% during the training and 69.05% for the test data. Considering the small sample size, this is a promising performance that could be further enhanced by expanding the dataset.

Table 9 documents the summary of the misclassification and error. This result indicates that, collectively, the error increased from the training data (23.81%) to the test data (30.95%) for all classes. Classifying over-dried timber had the best training result, with a 10.34% error, while acceptable class had the best test result with a 28.36% error.

Overall, classification could categorize timber pieces into three classes based on their *M_f_* with acceptable accuracy. This could be beneficial to wood manufacturing companies, as sawmills can apply this model to improve pre-and post-sorting strategies. The optimum breakpoints for the dry-sort-re-dry method could be accurately determined using the outcome of this classification approach. It is noteworthy that this research included some limitations, including relatively small sample size (42 boards per run) and single setpoint (*M_t_* = 12%). Therefore, future research should focus on a bigger sample size to improve the training and test performance of the model. Moreover, future studies should broaden the range of *M_f_* moisture prediction by selecting multiple *M_t_* for different drying runs. This study utilized the TreeNet gradient boosting model. Despite the proven effectiveness of tree-based ensemble models, future research can perform comparative studies to better reveal the performance of the selected model against other techniques, such as ANNs or support vector machines. While this research focused on *M_f_* between timber pieces, future studies may have to characterize and model *M_f_* within every single piece of timber (core and shells) [74] and casehardening [75]. Moreover, future studies may have to provide predictive and classifying models for drying defects, such as surface checks [76], internal checks (honeycombing) [77], and shape distortions [78]. Finally, future research should investigate the effectiveness of different NDE methods for the fast and reliable assessment of timber MC. Acoustic and ultrasound signals were shown to be sensitive to wood characteristics such as MC [79]. Additionally, the suitability of near-infrared (NIR) spectroscopy, as a widely used NDE method for wood characterization and monitoring [80,81,82,83], could be assessed for MC monitoring in kiln-dried timber at sawmills.

## 4. Conclusions

This research provided a holistic approach that considers selected wood indices and drying parameters in modeling moisture after kiln drying. Including the drying parameters in the model significantly improved the accuracy of the TreeNet, despite showing lower relative importance compared to the wood attributes. This finding emphasizes that a robust and accurate model should include not only wood attributes but also drying parameters. From a practical standpoint, *w_i_* had the highest correlation with *M_f_* among the input variables. This result was outstanding from a practical viewpoint, as weighing timber in sawmills is a fast and non-destructive test. The outcome of this research is an advanced step in optimizing drying schedules concerning final moisture variation. Classifying models are highly applicable to optimizing post-sorting strategies such as dry-sort-re-dry.

## Figures and Tables

**Table 1:** Nine drying batches of baby square western hemlock.

Run Number	Schedule	Conditioning	Storage	Name
1	Control (unmodified)	Yes	No	UN
2	Modified I	Yes	No	I_C_NS
3	Modified I	No	No	I_NC_NS
4	Modified II	No	No	II_NC_NS
5	Modified I	Yes	Yes	I_C_S
6	Modified I	No	Yes	I_NC_S
7	Modified II	Yes	Yes	II_C_S
8	Modified II	No	Yes	II_NC_S
9	Modified II	Yes	No	II_C_NS

**Table 2:** Control (unmodified) drying schedule used in industrial kilns in British Columbia sawmills. This schedule comprises eight time-based steps, one moisture-based step, and one conditioning step.

Step	Time (h)	Dry-Bulb Temperature (°C)	Wet-Bulb Temperature (°C)	Relative Humidity (%)	Equilibrium Moisture Content (%)
1	12	48.9	48.9	100.0	25.5
2	24	51.7	50.6	94.2	20.8
3	24	55.0	52.8	89.0	17.6
4	24	57.8	55.0	86.5	16.2
5	24	61.7	56.7	77.7	12.7
6	24	65.6	58.9	71.9	10.8
7	24	70.0	60.6	63.7	8.8
8	24	73.9	62.8	59.4	7.8
9	Till *M_f_* = 12%	77.8	65.0	55.7	7.0
10	12	71.7	66.7	79.4	12.3

**Table 3:** First modified schedule. This schedule comprises six time-based steps and one moisture-based step, followed by optional conditioning (step 8) and optional post-storage (step 9).

Step	Time (h)	Dry-Bulb Temperature (°C)	Wet-Bulb Temperature (°C)	Relative Humidity (%)	Equilibrium Moisture Content (%)
1	12	48.9	48.9	100.0	25.5
2	24	57.8	54.4	83.8	15.1
3	24	54.4	46.1	62.7	9.7
4	24	60.0	46.1	46.1	6.8
5	24	62.2	46.1	41.0	6.0
6	24	71.1	51.7	37.0	5.1
7	Till *M_f_* = 12%	78.8	54.4	30.1	4.1
8	12 (optional)	71.7	66.7	79.4	12.3
9	168 (optional)	20	16	65	12.3

**Table 4:** Second modified schedule. This schedule comprises five time-based steps and one moisture-based step, followed by optional conditioning (step 7) and optional post-storage (step 8).

Step	Time (h)	Dry-Bulb Temperature (°C)	Wet-Bulb Temperature (°C)	Relative Humidity (%)	Equilibrium Moisture Content (%)
1	12	48.9	48.9	100.0	25.5
2	24	62.8	60.6	89.8	17.2
3	24	68.3	64.4	83.2	13.9
4	24	71.1	64.4	73.1	10.7
5	24	79.4	64.4	50.4	6.2
6	Till *M_f_* = 12%	85.0	64.4	39.5	4.7
7	12 (optional)	71.7	66.7	79.4	12.3
8	168 (optional)	20	16	65	12.3

**Table 5:** The correlation between *M_f_* and initial wood indices in nine drying runs (abbreviated names of the drying schedules are defined in Table 1).

Drying Schedule	Correlation between Wood Indices		
	*M_i_* and *M_f_*	*w_i_* and *M_f_*	*ρ_b_* and *M_f_*
UN	0.15	0.40	0.18
I_C_NS	0.62	0.74	0.28
I_NC_NS	0.54	0.61	0.29
II_NC_NS	0.22	0.59	0.60
I_C_S	0.45	0.61	0.29
I_NC_S	0.47	0.75	0.50
II_C_S	0.53	0.61	0.15
II_NC_S	0.25	0.38	0.12
II_C_NS	0.37	0.55	0.28

**Table 6:** Model summary for predicting the *M_f_* using TreeNet including six inputs.

Statistics	Training (%)	Test (%)
R-squared (R^2^)	73.86	44.81
Root mean squared error (RMSE)	2.48	3.61
Mean squared error (MSE)	6.15	13.05
Mean absolute deviation (MAD)	1.68	2.43
Mean absolute percent error (MAPE)	0.12	0.17

**Table 7:** Model summary for predicting the *M_f_* using TreeNet including three inputs.

Statistics	Training (%)	Test (%)
R-squared (R^2^)	56.80	32.10
Root mean squared error (RMSE)	3.19	4.00
Mean squared error (MSE)	10.17	16.04
Mean absolute deviation (MAD)	2.33	2.92
Mean absolute percent error (MAPE)	0.17	0.21

**Table 8:** Confusion matrix and the summary of the classification with a random forest model.

Actual Class	Count	Predicted Training Class					Predicted Test Class		
		Over- Dried	Acceptable	Under-Dried	Correct (%)	Over- Dried	Acceptable	Under-Dried	Correct (%)
Over-dried	58	52	5	1	89.66	34	23	1	58.62
Acceptable	268	44	192	32	71.64	38	192	38	71.64
Under-dried	52	0	8	44	84.62	2	15	35	67.31
ALL	378	96	205	77	76.19	74	230	74	69.05

**Table 9:** Summary of misclassification and error for random forest model.

Actual Class	Count	Predicted Training Class		Predicted Test Class	
		Misclassed	Error (%)	Misclassed	Error (%)
Over-dried	58	6	10.34	24	41.38
Acceptable	268	76	28.36	76	28.36
Under-dried	52	8	15.38	17	32.69
ALL	378	90	23.81	117	30.95

## Data Availability

Not applicable.
